# Availability of Cancer Screening Across HIV Treatment Sites in the IeDEA Consortium

**DOI:** 10.1002/ijc.70503

**Published:** 2026-04-16

**Authors:** Rachael A. Pellegrino, Bryan E. Shepherd, Sanjay Pujari, Valeria Fink, Gad Murenzi, Miriam Nakalembe, Sally B. Coburn, Eliane Rohner, Antoine Jaquet, Caroline Lade, Brenda Crabtree Ramirez, Kathryn Anastos, Aggrey Semeere, Lesley S. Park, Limpho Mokone, Simon Boni, I Ketut Agus Somia, Emilia M. Jalil, Adebola Adedimeji, Omenge Orang'o, Michael J. Silverberg, Kumbirai Pise Quarter, Eugène Messou, Jeremy Ross, Eduardo Gotuzzo, Patricia Lelo, Helen Byakwaga, Oliver Ezechi, Jonathan Euvrard, Fernanda Maruri, Chad J. Achenbach, Jessica L. Castilho, International epidemiology Databases to Evaluate AIDS (IeDEA)

**Affiliations:** ^1^ Division of Infectious Diseases, Department of Medicine Vanderbilt University Medical Center Nashville Tennessee USA; ^2^ Department of Biostatistics Vanderbilt University Medical Center Nashville Tennessee USA; ^3^ Institute of Infectious Diseases Pune India; ^4^ Research Department Fundacion Huesped Buenos Aires Argentina; ^5^ Goodlife Access Kigali Rwanda; ^6^ University of Rwanda Kigali Rwanda; ^7^ Department Obstetrics and Gynecology Makerere University Kampala Uganda; ^8^ Infectious Diseases Institute, College of Health Sciences Makerere University Kampala Uganda; ^9^ Johns Hopkins University Baltimore Maryland USA; ^10^ Institute of Social and Preventive Medicine University of Bern Bern Switzerland; ^11^ University of Bordeaux, National Institute for Health and Medical Research (INSERM) UMR 1219, Research Institute for Sustainable Development (IRD) EMR 271, Bordeaux Population Health Centre Bordeaux France; ^12^ Gold Coast Sexual Health Service, Gold Coast Hospital and Health Service Southport Queensland Australia; ^13^ Departamento de Infectología Instituto Nacional de Ciencias Médicas y Nutrición Salvador Zubirán Mexico City Mexico; ^14^ Department of Medicine Albert Einstein College of Medicine Bronx New York USA; ^15^ Department of Epidemiology & Population Health Albert Einstein College of Medicine Bronx New York USA; ^16^ Stanford University School of Medicine Stanford California USA; ^17^ SolidarMed Mokhotlong Lesotho; ^18^ PAC‐CI Research Program Abidjan Côte d'Ivoire; ^19^ Faculty of Medicine Udayana University—Ngoerah Hospital Denpasar Bali Indonesia; ^20^ Instituto Nacional de Infectologia Evandro Chagas, FIOCRUZ Rio de Janeiro Brazil; ^21^ Department of Social Sciences and Health Policy Wake Forest University School of Medicine Winson Salem North Carolina USA; ^22^ Department of Implementation Science Wake Forest University School of Medicine Winson Salem North Carolina USA; ^23^ Department of Obstetrics and Gynaecology Aga Khan University Medical College Nairobi Kenya; ^24^ Kaiser Permanente Northern California Pleasanton California USA; ^25^ SolidarMed Masvingo Zimbabwe; ^26^ CePReF‐Aconda‐VS Abidjan Côte d'Ivoire; ^27^ TREAT Asia, amfAR—The Foundation for AIDS Research Bangkok Thailand; ^28^ Universidad Peruana Cayetano Heredia, Instituto de Medicina Tropical Alexander von Humboldt Lima Peru; ^29^ Kalembelembe Pediatric Hospital Kinshasa Democratic Republic of Congo; ^30^ Mbarara University of Science and Technology Mbarara Uganda; ^31^ Nigerian Institute of Medical Research Lagos Nigeria; ^32^ Centre for Integrated Data and Epidemiological Research, School of Public Health University of Cape Town Cape Town South Africa; ^33^ Department of Medicine and Robert J. Havey MD Institute for Global Health, Northwestern University Feinberg School of Medicine Chicago Illinois USA; ^34^ Department of Health Policy Vanderbilt University Medical Center Nashville Tennessee USA

**Keywords:** cancer, cancer screening, HIV

## Abstract

Cancer remains a leading cause of morbidity and mortality in people with HIV, and the importance of cancer screening increases as this population ages. In 2017, 2020, and 2023, cross‐sectional surveys were conducted at HIV care sites across 5 continents within the International epidemiology Databases to Evaluate AIDS (IeDEA) consortium. We described cancer screening availability in 2023 and over time for cervical and anal cancer screening using generalized estimating equations with a logit link function to account for site clustering, rurality, and country income. Of the 220 sites in the 2023 survey, 61% (134/220) reported cervical cancer screening by cytology or human papillomavirus (HPV) testing on‐site, and 88% (194/220) reported cervical cancer screening by any method, including visual inspection. At sites serving predominantly rural populations, 62% (29/47) exclusively performed screening by visual inspection. Overall, 23% (50/220) of sites performed cytology‐based anal cancer screening on‐site, and 16% (35/220) had availability of high‐resolution anoscopy, either on‐site or by referral. Screening for cancer of the liver, colon, lung, prostate, or breast (by imaging) were each available at less than 43% of sites. The odds of cervical cancer screening availability increased by 16% annually from 2017 through 2023 (aOR = 1.16, 95% CI: 1.07–1.27, *p* = 0.001), while the relative odds of anal cancer screening availability decreased by 9% annually (aOR = 0.91, 95% CI: 0.84–0.99, *p* = 0.023). Lack of trained staff was the most frequently reported barrier, followed by lack of equipment. Understanding current practices and capacity is essential for the continued integration of cancer prevention in HIV care.

## Abstract (highlights)

What's new?

In recent years, incorporating comprehensive cancer screening strategies into routine HIV care has gained growing attention. This survey of 220 sites providing HIV care across 41 countries shows that cervical cancer screening has increased in availability over time. Significant gaps, however, persist in the screening availability for cervical, anal, liver, colon, lung, and prostate cancer, with disparities observed by region, country income, and rurality of the population served. A lack of trained staff or equipment was the most frequently reported barriers. The findings highlight pressing needs for global cancer prevention in people living with HIV.

## Graphical abstract

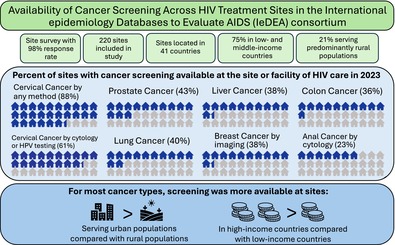

**Abbreviations**
aOR: adjusted odds ratio
CCASAnet: Caribbean, Central and South America network for HIV epidemiology
CI: confidence interval
CT: computed tomography
HBV: hepatitis B virus
HPV: human papillomavirus
HRA: high‐resolution anoscopy
IeDEA: International epidemiology Databases to Evaluate AIDS
NA‐ACCORD: North American AIDS Cohort Collaboration on Research and Design
PWH: people with HIV
REDCap: Research Electronic Data Capture
WHO: World Health Organization

## 1 Introduction

Cancer remains a leading cause of morbidity and mortality among people with HIV (PWH) around the world [1, 2, 3, 4, 5]. Immunodeficiency and coinfection with oncogenic viruses put PWH at elevated risk for certain cancers, such as cervical cancer, anal cancer, and hepatocellular carcinoma [1, 6, 7]. With the availability of antiretroviral therapy and sustained HIV virologic suppression, PWH are living longer and experiencing increased occurrence of noninfectious comorbidities, including aging‐related cancers [6, 8]. Among a US cohort in 2001–2015, lung cancer, liver cancer, and anal cancer contributed to 2.4%, 1.1%, and 0.6% of all deaths, respectively [6]. Additionally, these malignancies are often diagnosed at later stages and have a higher mortality in PWH compared with people without HIV [1, 6, 9].

Cancer screening has proven to be an effective and essential tool in reducing cancer‐associated morbidity and mortality through the early identification and treatment of cancer and pre‐cancerous lesions [6, 9]. Screening for cervical cancer has been critically important for PWH since the beginning of the epidemic, but screening for other cancers, including those associated with aging, has become increasingly necessary [10, 11]. For PWH, there are population‐specific screening recommendations for cervical and anal cancers, in addition to the general screening guidelines for colon, breast, lung, liver, and prostate cancer, which have mostly been studied and implemented in high‐income countries [9, 12, 13, 14]. In recent years, there has been increasing focus on methods for incorporating comprehensive cancer screening strategies into routine HIV care [15]. Understanding the availability of cancer screening and prevention within HIV treatment sites globally is critical for identifying and addressing gaps in service availability and for strengthening future efforts to integrate these services in HIV care. This is particularly important in low‐ and middle‐income countries (LMIC) and rural areas, where broader implementation of cancer prevention programs has faced challenges [16, 17, 18, 19].

The objectives of this study were to describe the availability and differences in current screening practices for cervical, anal, liver, breast, colon, prostate, and lung cancer at HIV treatment sites in the International epidemiology Databases to Evaluate AIDS (IeDEA) consortium. Additionally, for cervical and anal cancer screening, we examined the percentage of individuals receiving HIV care at sites without screening availability and trends in screening availability over time among IeDEA clinical sites. While the availability of screening does not necessarily reflect screening utilization or clinic practices, it is an important measure of screening implementation capacity.

## 2 Methods

The IeDEA consortium was established in 2006 and collects observational data representing people with or at risk for HIV from an open cohort of HIV treatment sites in 44 countries [20, 21, 22]. IeDEA data are organized into 7 geographical regions: the Asia‐Pacific; the Central, East, Southern, and West Africa regions; the Caribbean, Central and South America network for HIV epidemiology (CCASAnet); and the North American AIDS Cohort Collaboration on Research and Design (NA‐ACCORD) [20, 21, 22, 23]. The consortium has conducted periodic surveys of participating HIV treatment sites since 2009 in an effort to document clinic practices and service availability, with the most recent surveys conducted in 2017, 2020, and 2023 [24].

### 2.1 IeDEA Site Assessment Survey Design and Implementation

The 2023 IeDEA site assessment survey was designed using a consultative, concept‐driven approach similar to published methods for previous surveys [24]. All active sites contributing patient‐level data to IeDEA in 2023 were eligible to participate. In Southern Africa, where multiple small clinics contribute data as part of programmatic cohorts, a hybrid sampling strategy was used with 28 sites that had participated in prior surveys purposively sampled and 6 sites randomly selected from large programmatic cohorts. The survey was distributed to 240 sites on August 3, 2023, and closed on December 31, 2023.

The 2023 survey contained questions about site characteristics (location, type of facility, rurality, and age range of patient population served) and the availability of screening for cancer of the cervix, anus, liver, breast, colon, lung, and prostate. For each cancer screening method, the survey asked, “Which of the following cancer screenings were routinely performed during follow‐up visits for enrolled patients with HIV” and the response options were (1) Provided in the HIV clinic, (2) In the same health facility (but not at the HIV clinic), (3) Only offsite (referral), or (4) Not available. For screening of each cancer, the survey assessed the types of patients (male sex at birth, female sex at birth, patients in specific age groups, patients with specific risk factors, other patient groups, none) routinely screened and the clinic barriers that limited routine cancer screening being offered at the site.

The availability of various screening methods for each type of cancer was assessed in the 2023 survey (Table S1). For cervical cancer screening, the survey separately assessed screening by visual inspection (which could encompass visual inspection with acetic acid [VIA] and visual inspection with Lugol's iodine [VILI]), pap smear, and HPV testing (self‐collected or provider‐collected). For anal cancer screening, the survey separately assessed anal cytology and high‐resolution anoscopy (HRA). For liver cancer screening, screening by ultrasound and by other means were included separately. For breast cancer screening, the survey separately listed breast examination and imaging (including mammogram or ultrasound). For colon cancer screening, fecal occult blood tests and colonoscopy were grouped together. X‐ray and CT scan were listed together as examples for lung cancer screening and prostate cancer screening was described as by laboratory test, which would include prostate‐specific antigen.

The methods and implementation strategies for the 2017 and 2020 surveys were previously published, and sampling methods for sites from Southern Africa were similar to methods used in the 2023 survey [24, 25]. These surveys contained questions on the availability of cervical cancer screening by cytology or visual inspection and anal cancer screening by cytology. No questions on human papillomavirus (HPV) testing or barriers to cancer screening were included in the 2017 and 2020 surveys. The 2017 survey was administered to 255 sites between June and December 2017, and the 2020 survey was administered to 238 sites from September 2020 through February 2021.

For the 2017, 2020, and 2023 surveys, the questionnaire was distributed in both English and French as either an online or paper form. Surveys completed on paper were entered into the Research Electronic Data Capture (REDCap) web‐based data collection software by regional representatives or study staff members [26, 27]. Survey recipients were identified by regional and country coordinators as individuals who were knowledgeable about the clinical capacity and services at each site. Respondents were encouraged to consult with colleagues at their site, as needed, to respond to the survey questions. Completion of the survey was voluntary, and no incentives were provided. To facilitate completion, the research team sent reminders to sites and shared regular updates on regional survey progress, in addition to extending the survey deadline. All survey data were collected and managed using REDCap tools hosted at Vanderbilt University Medical Center.

### 2.2 Availability of Cancer Screening

We included data only from treatment sites that provided HIV care to individuals over 25 years of age. For the assessment of screening availability for each cancer, the primary outcome was a composite answer of availability of services in the HIV clinic or in the same health facility (considered jointly as “on‐site”) versus “Only off‐site (referral)” or “Not available.” For each participating site, the country's World Bank classification by income level was determined for the year that each survey was completed [28]. In the 2023 survey, rurality was assessed by the question “How would you describe the area of residence of the population served by this health facility's HIV clinic?” with the possible answers of (1) Predominantly urban, (2) Predominantly rural, (3) Mixed urban/rural. We summarized the availability of screening by region, rurality and World Bank country income designation using descriptive statistics. We used chi‐squared tests to assess screening differences by site‐level characteristics. Barriers to cancer screening in 2023 were also reported.

To estimate the potential screening needs for cervical and anal cancer, we matched sites participating in the 2023 survey with individual patient‐level data from IeDEA's regional cohorts, including patient age and sex. For sites with individual‐level data available, the clinic population at each site was defined as unique PWH with ≥ 1 clinic visit between January 1, 2021, and December 31, 2023. Individuals under 18 years of age at the start of follow‐up were excluded. This cross‐sectional analysis used routinely collected demographic data from PWH enrolled in observational HIV cohorts that participate in the IeDEA network. Eligibility for cervical cancer screening was defined as females between age 25 and 65 years old at their most recent visit during this time period [11, 29]. Eligibility for anal cancer screening was defined as men who have sex with men over age 35 years and all other PWH over age 45 years at their most recent visit during this time period [13]. Any availability of cancer screening was defined as screening available either in the HIV clinic, at the same facility, or by referral only.

### 2.3 Statistical Analysis

For cervical cancer, this analysis included any type of cervical cancer screening (including cytology, HPV testing, or visual inspection) as well as the availability of cervical cancer screening by cytology or HPV testing (excluding visual inspection). For anal cancer screening, separate analyses assessed the availability of anal cytology and HRA. The percentage of individuals receiving care at a site without any available anal or cervical cancer screening was calculated as the number of screening‐eligible PWH at clinics without any available screening divided by the total number of screening‐eligible PWH. We reported the variability in the size of the population eligible for screening and availability of screening by region and by country‐income. Incomplete individual‐level data on sexual orientation may lead to an underestimation of the number of individuals eligible for anal cancer screening. Therefore, we conducted a sensitivity analysis to estimate the number of individuals who may have been misclassified as not eligible for anal cancer screening because their sexual orientation was not known (men ages 35–45 years with unknown sexual orientation).

### 2.4 Cervical and Anal Cancer Screening Availability Over Time

Using the responses from the 2017, 2020, and 2023 surveys, we evaluated the change in on‐site availability of cervical cancer and anal cancer screening at sites providing care to adult patients over this time period. For these analyses we assessed cervical cancer screening by visual inspection or cytology and anal cancer screening by cytology.

### 2.5 Statistical Analysis

We used generalized estimating equations with a logit link function and an independent working correlation structure to account for clustering of responses by sites that had participated in multiple surveys. Additionally, we adjusted for rurality and country income based on the World Bank classification. Over all three surveys, there was only one response with missing information on cancer screening availability, and this response was excluded. We also conducted a restricted analysis to assess only the 126 sites that had completed the survey at all three time points. The attrition and inclusion of sites over the survey years is shown in Figure S1.

## 3 Results

**FIGURE 1:**
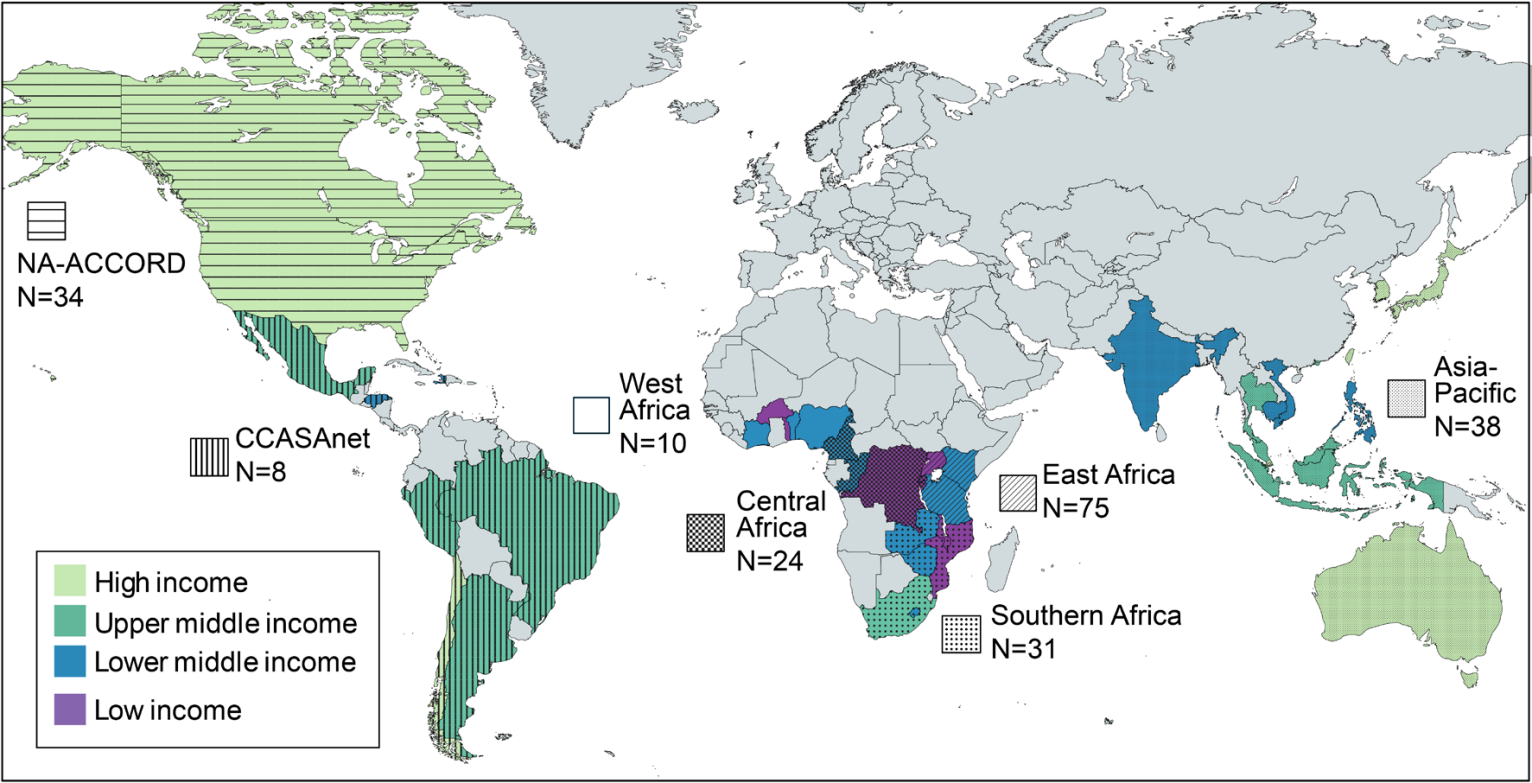
Geographic distribution of the countries represented by the 220 sites in the IeDEA network participating in the 2023 site assessment survey. The World Bank country income designation is identified by color and the IeDEA region is identified by pattern, which includes NA‐ACCORD (Canada, US), CCASAnet (Argentina, Brazil, Chile, Haiti, Honduras, Mexico, Peru), West Africa (Benin, Burkina Faso, Cote d'Ivoire, Nigeria, Togo), Central Africa (Burundi, Cameroon, Dem. Rep. Congo, Rep. Congo, Rwanda), East Africa (Kenya, Tanzania, Uganda), Southern Africa (Lesotho, Malawi, Mozambique, South Africa, Zambia, Zimbabwe), and the Asia‐Pacific (Australia, Cambodia, Hong Kong SAR, India, Indonesia, Japan, Rep. Korea, Malaysia, Philippines, Singapore, Taiwan, Thailand, Vietnam).

Of the 240 eligible sites in the 2023 survey, 235 (98%) responded. Of sites completing the survey, 220 provided care for individuals over age 25 and were included in the analysis. In 2023, sites included in this study represented 41 different countries (Figure 1) [30], 75% were in LMIC, 21% served a predominantly rural population, and 62% identified as health centers (Table 1). In 2020, 227 (95%) of the 238 eligible sites completed the survey, and in 2017, 239 (94%) of the 255 eligible sites completed the survey. Of the 220 responses included from the 2023 survey, 173 (79%) were completed by clinical staff (136 by clinicians, 37 by nurses), and 37 (17%) were completed by site managers.

**TABLE 1:** Characteristics of HIV treatment sites that completed the IeDEA site assessment survey in 2017, 2020, and 2023.

	2017 survey (*n* = 205)	2020 survey (*n* = 204)	2023 survey (*n* = 220)
IeDEA region			
Asia‐Pacific	43 (21.0%)	40 (19.6%)	38 (17.3%)
CCASAnet	14 (6.8%)	8 (3.9%)	8 (3.6%)
Central Africa	19 (9.3%)	21 (10.3%)	24 (10.9%)
East Africa	44 (21.5%)	73 (35.8%)	75 (34.1%)
NA‐ACCORD	41 (20.0%)	30 (14.7%)	34 (15.5%)
Southern Africa	35 (17.1%)	25 (12.3%)	31 (14.1%)
West Africa	9 (4.4%)	7 (3.4%)	10 (4.5%)
Residence of population served			
Urban	123 (60.0%)	73 (35.8%)	94 (42.7%)
Mixed Urban/Rural	36 (17.6%)	88 (43.1%)	79 (35.9%)
Rural	46 (22.4%)	43 (21.1%)	47 (21.4%)
Facility level of care			
Health center	99 (48.3%)	119 (58.3%)	131 (59.5%)
District hospital	17 (8.3%)	15 (7.4%)	16 (7.3%)
Regional, provincial or university hospital	76 (37.1%)	62 (30.4%)	65 (29.5%)
Unknown	13 (6.3%)	8 (3.9%)	8 (3.6%)
World Bank income designation			
High income	67 (32.7%)	52 (25.5%)	55 (25.0%)
Upper middle income	28 (13.7%)	25 (12.3%)	28 (12.7%)
Lower middle income	51 (24.9%)	79 (38.7%)	82 (37.3%)
Low income	59 (28.8%)	48 (23.5%)	55 (25.0%)

### 3.1 Cervical Cancer Screening

In 2023, 61% (134/220) of sites reported routinely performing cervical cancer screening by cytology or HPV testing on‐site and 88% (194/220) of sites reported cervical cancer screening by any method (visual inspection, cytology, or HPV testing) on‐site. Cervical cancer screening by visual inspection was available at 81% (179/220) of sites, while testing with cytology was performed at 54% (118/220) of sites, and HPV testing at 45% (98/220) of sites (Table 2). At sites serving predominantly rural populations, 32% (15/47) had cytology available, compared with 68% (64/94) of sites serving predominantly urban populations. Similarly, HPV testing was available at 64% (60/94) of urban sites compared with 9% (4/47) of rural sites. Of sites serving rural populations, 62% (29/47) of sites exclusively performed screening by visual inspection. In high‐income countries, cytology and HPV testing were widely available at 87% (48/55) and 91% (50/55) of sites, respectively, which was statistically higher than in low‐income countries where cytology and HPV testing were available at 46% (25/55) and 29% (16/55) of sites, respectively. While the primary analysis assessed the availability of screening on‐site (including in the HIV clinic and at the same healthcare facility), Table S2 shows the on‐site availability data for cervical and anal cancer screening methods further separated by “HIV clinic” and “same facility.”

**TABLE 2:** Site‐level characteristics and reported on‐site availability of cancer screening services at HIV treatment sites in the IeDEA consortium, 2023 (*n* = 220). Count and row percentages shown. Statistically different (*p* < 0.05) availability of each type of cancer screening was seen by residence of population served and World Bank income designation, except for screening for breast cancer by provider exam.

	Cervical cancer			Anal cancer			Liver cancer			Breast cancer		
	Visual	Cytology	HPV^a^	Cytology	HRA	Imaging^b^	Other^c^	Exam	Imaging^d^	Colon cancer^e^	Lung cancer^f^	Prostate cancer^g^
Overall (*n* = 220)	179 (81.4%)	118 (53.6%)	98 (44.5%)	50 (22.7%)	35 (15.9%)	83 (37.7%)	77 (35.0%)	179 (81.4%)	83 (37.7%)	80 (36.4%)	88 (40.0%)	94 (42.7%)
IeDEA region												
Asia‐Pacific (*n* = 38)	22 (57.9%)	25 (65.8%)	28 (73.7%)	10 (26.3%)	10 (26.3%)	23 (60.5%)	25 (65.8%)	25 (65.8%)	20 (52.6%)	22 (57.9%)	21 (55.3%)	25 (65.8%)
CCASAnet (*n* = 8)	5 (62.5%)	5 (62.5%)	2 (25.0%)	3 (37.5%)	5 (62.5%)	5 (62.5%)	5 (62.5%)	6 (75.0%)	4 (50.0%)	5 (62.5%)	5 (62.5%)	4 (50.0%)
Central Africa (*n* = 24)	16 (66.7%)	12 (50.0%)	9 (37.5%)	1 (4.2%)	0	6 (25.0%)	5 (20.8%)	23 (95.8%)	6 (25.0%)	5 (20.8%)	8 (33.3%)	5 (20.8%)
East Africa (*n* = 75)	75 (100%)	23 (30.7%)	13 (17.3%)	3 (4.0%)	1 (1.3%)	13 (17.3%)	7 (9.3%)	59 (78.7%)	8 (10.7%)	6 (8.0%)	11 (14.7%)	11 (14.7%)
NA‐ACCORD (*n* = 34)	30 (88.2%)	33 (97.1%)	32 (94.1%)	26 (76.5%)	18 (52.9%)	27 (79.4%)	27 (79.4%)	32 (94.1%)	29 (85.3%)	29 (85.3%)	27 (79.4%)	32 (94.1%)
Southern Africa (*n* = 31)	24 (77.4%)	19 (61.3%)	9 (29.0%)	7 (22.6%)	1 (3.2%)	6 (19.4%)	6 (19.4%)	28 (90.3%)	12 (38.7%)	11 (35.5%)	13 (41.9%)	14 (45.2%)
West Africa (*n* = 10)	7 (70.0%)	1 (10.0%)	5 (50.0%)	0	0	3 (30.0%)	2 (20.0%)	6 (60.0%)	4 (40.0%)	2 (20.0%)	3 (30.0%)	3 (30.0%)
Residence of population served												
Urban (*n* = 94)	70 (74.5%)	64 (68.1%)	60 (63.8%)	35 (37.2%)	26 (27.7%)	50 (53.2%)	50 (53.2%)	76 (80.9%)	54 (57.4%)	53 (56.4%)	54 (57.4%)	57 (60.6%)
Mixed Urban/Rural (*n* = 79)	66 (83.5%)	39 (49.4%)	34 (43.0%)	13 (16.5%)	8 (10.1%)	28 (35.4%)	24 (30.4%)	68 (86.1%)	25 (31.6%)	23 (29.1%)	29 (36.7%)	31 (39.2%)
Rural (*n* = 47)	43 (91.5%)	15 (31.9%)	4 (8.5%)	2 (4.3%)	1 (2.1%)	5 (10.6%)	3 (6.4%)	35 (74.5%)	4 (8.5%)	4 (8.5%)	5 (10.6%)	6 (12.8%)
World Bank income designation												
High income (*n* = 55)	45 (81.8%)	48 (87.3%)	50 (90.9%)	31 (56.4%)	24 (43.6%)	40 (72.7%)	42 (76.4%)	47 (85.5%)	40 (72.7%)	41 (74.5%)	38 (69.1%)	48 (87.3%)
Upper middle income (*n* = 28)	17 (60.7%)	23 (82.1%)	15 (53.6%)	11 (39.3%)	8 (28.6%)	14 (50.0%)	14 (50.0%)	25 (89.3%)	17 (60.7%)	18 (64.3%)	20 (71.4%)	22 (78.6%)
Lower middle income (*n* = 82)	67 (81.7%)	22 (26.8%)	17 (20.7%)	7 (8.5%)	3 (3.7%)	20 (24.4%)	14 (17.1%)	65 (79.3%)	19 (23.2%)	14 (17.1%)	20 (24.4%)	17 (20.7%)
Low income (*n* = 55)	50 (90.9%)	25 (45.5%)	16 (29.1%)	1 (1.8%)	0	9 (16.4%)	7 (12.7%)	42 (76.4%)	7 (12.7%)	7 (12.7%)	10 (18.2%)	7 (12.7%)

Abbreviation: HRA, high resolution anoscopy.

^a^ Molecular cervical HPV testing by self‐collection or provider‐collected.

^b^ Liver cancer screening by ultrasound.

^c^ Other liver cancer screening tests include CT scan or serum alpha‐fetoprotein measurements.

^d^ Imaging for breast cancer screening included mammography or ultrasound.

^e^ Colon cancer screening included fecal occult blood tests or colonoscopy.

^f^ Lung cancer screening included by X‐ray and CT scan.

^g^ Prostate cancer screening by laboratory test.

**FIGURE 2:**
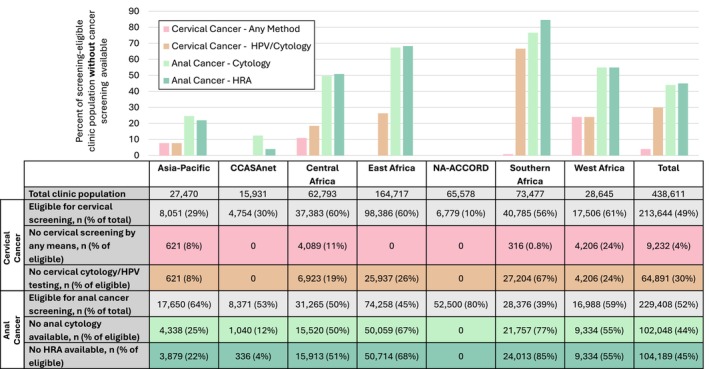
Estimated number and percentage of individuals eligible for cervical and anal cancer screening and of those who receive care at sites where each cancer screening method is not available either on‐site or by referral in 2023, by IeDEA region. In total, 165 completed site surveys could be linked to individual‐level data in IeDEA's regional cohorts for this analysis: Asia‐Pacific (*n* = 31), CCASAnet (*n* = 6), Central Africa (*n* = 24), East Africa (*n* = 65), NA‐ACCORD (*n* = 15), Southern Africa (*n* = 17), West Africa (*n* = 7). Countries included in each IeDEA region are described in Figure 1.

Of the 220 sites, 165 (75%) sites could be linked to individual‐level data in IeDEA's regional cohorts, with an estimated patient population of 438,611 PWH with at least one clinic visit between 2021 and 2023. By country income designation, 71,414 PWH resided in high‐income countries, 18,715 in upper middle‐income countries, 173,678 in lower middle‐income countries, and 174,804 in low‐income countries. These data were used to estimate the percentage of individuals without access to cervical or anal cancer screening at the site of their HIV care or through referral. In total, 213,644 PWH (49% of the total patient population) were eligible for cervical cancer screening (Figure 2). This ranged from 10% of the adult clinic population in NA‐ACCORD to 61% of the adult clinic population in West Africa, which reflects the sex and age distribution of these cohorts. Additionally, by country income, this ranged from 10% (7272/71,414) of the clinic population in high‐income countries to 57% (99,978/174,804) in low‐income countries. Of all individuals eligible for cervical cancer screening seen at a participating site, 4% (9232 PWH total) received HIV care at a site without any availability of cervical cancer screening by any method on‐ or off‐site. When looking only at cervical cancer screening using the World Health Organization‐recommended methods of HPV testing or cytology, 30% (64,891 PWH) did not have access, which ranged from 67% of individuals without access in Southern Africa to none in CCASAnet and NA‐ACCORD. By country income, this ranged from 36% (35,887/99,978) of screening‐eligible PWH in low‐income countries to none in high‐income countries.

Cervical cancer screening by visual inspection or cytology was available on‐site at 73% (149/204) of sites completing the 2017 survey, 75% (153/204) of sites completing the 2020 survey, and 86% (190/220) of sites completing the 2023 survey. After adjusting for country income‐level and rurality, the odds of cervical cancer screening availability multiplicatively increased by 16% annually from 2017 through 2023 (adjusted odds ratio [aOR] = 1.16, 95% CI: 1.07–1.27, *p* = 0.001) (Table S3). When the analysis was restricted to those sites that completed the survey at every time point, the odds of cervical screening availability increased by 3% annually, but this was not statistically significant (aOR = 1.03, 95% CI: 0.93–1.16, *p* = 0.554).

### 3.2 Anal Cancer Screening

Anal cytology was available on‐site at 23% (50/220) of all sites, at 4% (2/47) of sites serving rural populations, and at 2% (1/55) of sites in low‐income countries (Table 2). HRA was available on‐site at 16% (35/220) of sites, but at 2% (1/47) of sites serving rural populations. No sites in low‐income countries reported availability of HRA. Abnormal anal cytology results should be followed up by an exam with HRA, and nearly all (45/50) sites with anal cytology on‐site also reported access to HRA either on‐site (*n* = 31) or by referral (*n* = 14) [13]. When asked about which patients were screened for anal cancer in 2023, 19% (41/220) of sites reported routinely screening males, 9% (19/220) of sites reported routinely screening females, and 32% (70/220) reported screening patients with specific risk factors not further defined. Additional details on the location of available screening (HIV clinic, same facility, off‐site) can be found in Table S2.

From the individual‐level data of 165 sites, 229,408 PWH (52% of the patient population) were eligible for anal cancer screening (Figure 2). This ranged from 39% of the clinic population in Southern Africa to 80% of the clinic population in NA‐ACCORD. By country income designation, 79% (56,256/71,414) of the clinic population in high‐income countries were eligible for anal cancer screening compared to 40% (70,207/174,804) of the clinic population in low‐income countries. Of individuals eligible for anal cancer screening at a participating site, 44% (102,048 PWH total) received HIV care at a site without any availability of cytology‐based anal cancer screening either on‐ or off‐site, which ranged from 77% without access in Southern Africa to none in NA‐ACCORD. In high‐income countries, 3% (1473/56,256) of the screening‐eligible clinic population received care at a site without anal cytology compared to 79% (55,662/70,207) in low‐income countries. For HRA, 45% (104,189 PWH) of screening‐eligible individuals at IeDEA sites included in the site assessment survey received care at a site without availability of this service, which ranged from 85% without access in Southern Africa to none in NA‐ACCORD (Figure 2). By country income, this ranged from 1% (598/56,256) of PWH in high‐income countries to 81% (57,179/70,207) in low‐income countries. In a sensitivity analysis exploring those with “unknown” sexual orientation, we found that there were potentially 20,672 additional men who would be eligible for anal cancer screening, if their sexual orientation was known to be MSM. This would put the range of eligible PWH between 229,408 PWH (52% of the patient population) and 250,080 (57% of the patient population).

Anal cancer screening by cytology was available on site at 36% (73/204) of sites completing the 2017 survey, 29% (60/204) of sites completing the 2020 survey, and 22% (50/220) of sites completing the 2023 survey. After adjusting for country income‐level, rurality and clustering by site, the relative odds of anal cancer screening availability decreased by 9% annually between these periods (aOR = 0.91, 95% CI: 0.84–0.99, *p* = 0.023). When the analysis was restricted to those sites that completed the survey at every time point, the relative odds decreased annually by 8%, but this was not statistically significant (aOR = 0.92, 95% CI: 0.83–1.02, *p* = 0.126).

### 3.3 Other Cancer Screening

Screening for liver cancer with ultrasound was available in 2023 at 38% (83/220) of sites and screening with other tests, such as computed tomography (CT) scan or serum alpha‐fetoprotein was available at 35% (77/22) of sites (Table 2). Screening for colon cancer (by fecal occult blood test or colonoscopy), lung cancer (by X‐ray or CT scan), and prostate cancer (by laboratory test) was available at 36% (80/220), 40% (88/220), and 43% (94/220) of sites, respectively. Breast cancer screening by physical exam was available at 81% (179/220) of sites, while breast cancer screening through mammography or ultrasound was available at 38% (83/220) of sites. Statistically lower availability of each type of cancer screening was seen in sites serving rural populations and in low‐income countries, except for screening for breast cancer by provider exam (Table 2).

### 3.4 Barriers to Cancer Screening

**FIGURE 3:**
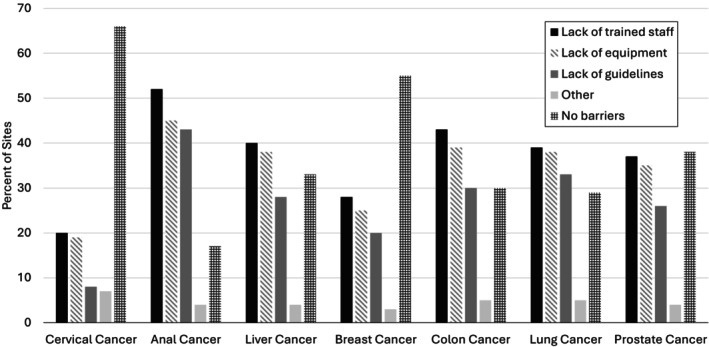
Clinic‐reported barriers limiting on‐site screening availability by cancer type, 2023. Respondents could select all answers that apply.

For each type of cancer screening, lack of trained staff was the most frequently reported clinic barrier to offering a service on‐site, followed by lack of equipment and lack of clinical guidelines (Figure 3). For most types of cancer screening, Southern Africa had the highest percentage of clinics reporting lack of trained staff, while East Africa had the highest percentage of clinics reporting lack of equipment as a barrier. Both barriers were reported the least by clinics in NA‐ACCORD.

## 4 Discussion

In this survey of 220 sites providing HIV care across 41 countries, we assessed the availability and barriers to cancer screening. Our analysis found that cervical cancer screening increased in availability over time, but significant gaps exist in the availability of screening for anal, liver, colon, lung, and prostate cancer for PWH at IeDEA sites. For almost all cancer screening, disparities in availability were observed at sites serving predominantly rural populations and sites located in low‐income countries. Additionally, the size of the screening‐eligible population and the percentage of those without access to anal and cervical cancer screening varied widely by region.

This analysis showed that cervical cancer screening, which has long been the target of international campaigns and funding, was available in some form at almost all HIV clinical sites, and this availability increased over time [10, 12]. Cervical cancer screening is one of the three components of the 2020 World Health Organization (WHO) global strategy to eliminate cervical cancer and has been integrated into comprehensive HIV care for women who access care at President's Emergency Plan for AIDS Relief (PEPFAR)‐supported facilities since 2018 [10, 11, 12]. The 2021 WHO guidelines recommend cervical cancer screening for women with HIV starting at age 25, and the suggested approach is the use of HPV DNA as the primary screening test with or without triage by another method (such as visual inspection with acetic acid or cytology) after a positive HPV test [12]. If sites are performing quality‐assured cytology, the recommendations are to continue this method until HPV testing is operational, but programs using visual inspection with acetic acid should be transitioned rapidly given the inherent challenges with quality assurance [12]. Less than half of sites in our study reported offering HPV testing in 2023, with availability concentrated in urban areas and high‐income countries. Screening volume, financial constraints, and insufficient infrastructure may be barriers to widespread implementation of this method. Visual inspection remained the predominant screening method in many resource‐limited areas. While this is no longer a WHO recommended screening method, it remains a recommended option for cervical cancer screening in some country‐specific guidelines [11, 31]. A meta‐analysis found that new diagnoses of cervical cancer occurring among women with HIV remain high in Southern Africa, where 63.8% of women with cervical cancer were also living with HIV [32]. In our study population, PWH attending clinics in Southern Africa had the highest number of individuals receiving care at sites without availability of cervical cancer screening by cytology or HPV testing, presenting an opportunity and urgency for the expansion of screening resources in these settings [12].

Similar to cervical cancer screening, anal cancer screening aims to detect and treat HPV‐associated precancerous lesions to prevent their progression to anal cancer [13]. Recommendations for anal cancer screening in PWH have evolved rapidly in recent years, and implementation of screening may be lagging at many sites included in this study for this reason. Following the publication of the ANCHOR trial results in 2022, which demonstrated the benefits of treating anal precancerous lesions to reduce the progression to anal cancer, multiple societies in the United States, Europe, and Brazil released anal cancer screening guidelines [13, 33, 34]. Recommended screening methods include cytology and HPV testing, which can be done alone, as co‐testing, or with one method followed by use of the other as triage for further evaluation [13, 35]. Regardless of the screening method used, abnormal results should be evaluated with HRA, which requires specialized equipment and training [33, 36]. While most sites in our study lacked on‐site and off‐site access to HRA, it is reassuring that 90% of those who offer on‐site anal cytology testing had HRA available for follow‐up. The availability of HRA is a key barrier to the expansion of anal cancer screening programs. Of programs that offered anal cancer screening, this service was generally restricted to men or individuals with specific risk factors. The observed decrease in anal cancer screening was seen prior to the publication of the most recent guidelines and may also be reflective of decreasing availability of cytology, funding changes, or limited HRA services. Additionally, we did not capture data on anal HPV testing, so any increases in this modality would not have been captured by this analysis. Anal cancer screening may not be available to PWH outside of their HIV care, and continued efforts in capacity‐building are necessary for the expansion and integration of screening services as guidelines are developed and implemented. There are also opportunities for the development of novel point‐of‐care devices with capabilities for high‐resolution imaging, easy sample collection, and rapid HPV testing. These emerging tools for anal cancer screening have the potential to further refine risk stratification and to reduce unnecessary procedures.

The availability of other cancer screenings varied widely by region, rurality, and country income. There were gaps in screening for liver, breast, colon, lung, and prostate cancer particularly in low‐income countries and sites serving rural populations. The exception to this was screening for breast cancer by provider exam, which is a low‐cost screening test and was widely available. While the WHO position paper recommends organized, population‐based mammography screening programs in settings with healthcare system capacity, clinical breast examination is noted as a promising approach in low‐resource settings though it does not have the appropriate level of evidence to support its full implementation [37, 38]. Screening recommendations for breast, colon, prostate, and lung cancer in PWH are similar to those of the general population and efforts to reduce financial and infrastructural barriers may increase integration of these services into HIV care [9]. In PWH, screening for liver cancer is targeted specifically to those with cirrhosis and/or hepatitis B virus (HBV) co‐infection, which is estimated to affect 7.6% of PWH with the greatest burden in Africa [39]. HIV/HBV co‐infection is often managed by HIV treatment sites, but liver cancer screening was only available at one third of sites and consolidating these services may increase uptake [9].

Integration of cancer screening into existing HIV care infrastructure provides the opportunity to decrease cancer‐associated morbidity and mortality [9]. This has been successfully done for cervical cancer screening in many places and there is room for improvement in screening for other types of cancer [10]. Successful cancer screening programs require resources for training, supportive supervision and guidance for difficult cases, and quality assurance evaluations [10]. Lack of trained staff and lack of equipment were identified as the most frequent barriers to each type of cancer screening in our study. Other barriers to cancer screening implementation that have been previously identified include lack of centralized screening databases, funding required for screening, and the infrastructure to confirm and treat abnormal findings [31]. Many of these barriers have been seen in cancer screening implementation efforts in both the general healthcare system and in the HIV clinic setting [40, 41, 42]. The funding for comprehensive HIV care, including cancer screening, is threatened in many parts of the world, putting current services and the necessary expansion in jeopardy [43].

There are multiple limitations to consider in the interpretation of these results. Sites within IeDEA may not have been representative of all HIV treatment sites in a country or region and this representativeness may vary by IeDEA region. The sites within IeDEA may also have been better resourced and provide more specialized care than other clinics, leading to overestimating the availability of services. The main analysis assessed cancer screening availability at the site and/or facility of HIV care, but this availability may not reflect actual utilization of or access to cancer screening by PWH. Though cancer screening may be reported as available, underutilization may occur for multiple reasons. Similarly, when examining the changes in cervical and anal cancer screening availability between the 2017 and 2023 surveys, we were only able to measure the change in the proportion of sites with services available, which did not capture if a site had increased or decreased screening capacity or patient utilization across this period. Furthermore, for each year of the site survey, the specific sites that were included and that responded were variable. In estimating the percentage of individuals seen at sites without screening available, not all sites that completed the survey were able to be linked to individual‐level data and these linked populations may not be representative of the clinic population of each respective country or region. Data on sexual orientation is limited in some regions, which may lead to an underestimation of the clinic population eligible for anal cancer screening. Additionally, we used international guidelines to determine the population eligible for cervical or anal cancer screening, but this may not reflect the national or local guidelines that influenced practice.

Finally, there are limitations in the data collected for each type of cancer screening. For anal cancer screening, we do not have data on the availability of anal HPV testing, digital anal rectal examination (DARE), or standard anoscopy (which may have been used as follow up for abnormal results). By not capturing DARE or HPV testing, we may be underestimating the overall screening availability. Additionally, we do not have data on HRA availability over time, which limits the ability to assess capacity for screening follow up during this time. For lung cancer screening, examples were listed as either CT scan or X‐ray, but X‐ray is no longer a recommended screening modality in most guidelines. Guidelines for prostate cancer have evolved over recent years and include shared decision making in many cases, which may affect screening availability.

## 5 Conclusions

This study showed that screening for cervical cancer has become increasingly available at IeDEA sites, but the availability of other cancer screenings remained limited with disparities by region, country income, and rurality of the population served. There is a continued need for the expansion of these services. Future studies should explore the modifiable barriers to the successful integration and expansion of cancer screening services to identify and refine implementation strategies.

## Author Contributions

**Rachael A. Pellegrino:** conceptualization, methodology, investigation, formal analysis, writing – original draft, writing – review and editing. **Bryan E. Shepherd:** conceptualization, methodology, investigation, formal analysis, writing – review and editing. **Sanjay Pujari:** data curation, resources, writing – review and editing. **Valeria Fink:** conceptualization, methodology, writing – review and editing. **Gad Murenzi:** conceptualization, methodology, writing – review and editing. **Miriam Nakalembe:** data curation, resources, writing – review and editing. **Sally B. Coburn:** data curation, resources, writing – review and editing. **Eliane Rohner:** conceptualization, methodology, writing – review and editing. **Antoine Jaquet:** conceptualization, methodology, writing – review and editing. **Caroline Lade:** data curation, resources, writing – review and editing. **Brenda Crabtree Ramirez:** conceptualization, methodology, writing – review and editing. **Kathryn Anastos:** conceptualization, methodology, writing – review and editing. **Aggrey Semeere:** conceptualization, methodology, writing – review and editing. **Lesley S. Park:** data curation, resources, writing – review and editing. **Limpho Mokone:** data curation, resources, writing – review and editing. **Simon Boni:** conceptualization, methodology, writing – review and editing. **I Ketut Agus Somia:** data curation, resources, writing – review and editing. **Emilia M. Jalil:** data curation, resources, writing – review and editing. **Adebola Adedimeji:** conceptualization, methodology, writing – review and editing. **Omenge Orang'o:** conceptualization, methodology, writing – review and editing. **Michael J. Silverberg:** data curation, resources, writing – review and editing. **Kumbirai Pise Quarter:** data curation, resources, writing – review and editing. **Eugène Messou:** data curation, resources, writing – review and editing. **Jeremy Ross:** conceptualization, methodology, writing – review and editing. **Eduardo Gotuzzo:** data curation, resources, writing – review and editing. **Patricia Lelo:** data curation, resources, writing – review and editing. **Helen Byakwaga:** data curation, resources, writing – review and editing. **Oliver Ezechi:** data curation, resources, writing – review and editing. **Jonathan Euvrard:** data curation, resources, writing – review and editing. **Fernanda Maruri:** data curation, resources, writing – review and editing. **Chad J. Achenbach:** conceptualization, methodology, writing – review and editing. **Jessica L. Castilho:** conceptualization, methodology, investigation, formal analysis, writing – original draft, writing – review and editing.

## Funding

This work was supported by the National Center for Advancing Translational Sciences, UL1 TR000445; National Institutes of Health, 5K12CA090625‐25, 5T32AI007474‐29, R24AI124872, U01AI069907, U01AI069911, U01AI069918, U01AI069919, U01AI069923, U01AI069924, U01AI096299.

## Ethics Statement

Ethical clearance for the study was obtained by local institutional review boards (IRBs) as well as the Vanderbilt University Medical Center IRB; a waiver of consent was obtained for participation in this analysis. Each IeDEA‐wide site assessment survey was reviewed by the Vanderbilt University Institutional Review Board and given a non‐research status per 45 CFR §46.102(l) (IRB#161017, IRB#200013, IRB#230843). All study activities complied with the Declaration of Helsinki.

## Conflicts of Interest

E.M.J. would like to disclose participation in a scientific committee (concluded) and research grant (concluded) from MSD. The remaining authors declare no conflicts of interest.

## Supporting information


**Table S1:** Questions 1, 2, and 15 of the IeDEA 2023 site assessment survey.
**Table S2:** Site‐level characteristics and reported availability of cancer screening services at HIV treatment sites either at the HIV clinic, in the same facility, or off‐site in the IeDEA consortium, 2023 (*n* = 220). Count and row percentages shown.
**Table S3:** Adjusted odds of cervical and anal cancer screening from 2017 through 2023 using generalized estimating equations with a logit link function to account for site clustering, rurality, and country income‐level by World Bank income designation.
**Figure S1:** Flow diagram of survey respondents for each survey. Survey 1 was conducted in 2017, survey 2 in 2020 and survey 3 in 2023.

## Data Availability

Data are available via IeDEA (www.iedea.org). Further information is available from the corresponding author upon request.
