# Supplementary material for: Availability of Cancer Screening Across HIV Treatment Sites in the IeDEA Consortium
Source: Int J Cancer. 2026 Apr 16;159(6):1477–89. doi: 10.1002/ijc.70503 (PMC13397155; doi:10.1002/ijc.70503)

Supplementary Materials

Availability of Cancer Screening Across HIV Treatment Sites in the IeDEA Consortium

Rachael A. Pellegrino, Bryan E. Shepherd, Sanjay Pujari, Valeria Fink, Gad Murenzi, Miriam Nakalembe, Sally B. Coburn, Eliane Rohner, Antoine Jaquet, Caroline Lade, Brenda Crabtree Ramirez, Kathryn Anastos, Aggrey Semeere, Lesley S. Park, Limpho Mokone, Simon Boni, I Ketut Agus Somia, Emilia M. Jalil, Adebola Adedimeji, Omenge Orang'o, Michael J. Silverberg, Kumbirai Pise Quarter, Eugène Messou, Jeremy Ross, Eduardo Gotuzzo, Patricia Lelo, Helen Byakwaga, Oliver Ezechi, Jonathan Euvrard, Fernanda Maruri, Chad J. Achenbach, Jessica L. Castilho on behalf of International epidemiology Databases to Evaluate AIDS (IeDEA)

Table of Contents

Supplementary Table 1……………………………………………………………...page 2

Supplementary Table 2……………………………………………………………...page 4

Supplementary Table 3..…………………………………………………………….page 6

Supplementary Figure 1 ..…...………………………………………………………...page 6

Supplementary Table 1: Questions 1, 2, and 15 of the IeDEA 2023 Site Assessment Survey

IeDEA 2023 Site Assessment Survey

The purpose of this survey is to learn about the clinical and support services provided to patients who are enrolled in HIV care at this health facility. This survey is being conducted at all health facilities participating in the International Epidemiology Databases to Evaluate AIDS (IeDEA) network.

- This survey is intended to be completed by staff who have in-depth knowledge about the care and services provided to adult and pediatric patients in the HIV clinic or within the health facility or institution.
- Most questions refer to care and services provided within the HIV clinic. If your health facility does not have a dedicated clinic for HIV care and treatment, please answer for the facility overall, regardless of what unit(s) serves these patients. If your health facility has multiple HIV care and treatment clinics that serve different patient groups, please report on the services provided for adult patients living with HIV, unless otherwise indicated.
- A few questions in this survey may require consultation with staff in other units, such as laboratory and pharmacy departments.
- Remember that there are no incorrect answers to this survey. Your feedback on day-to-day service delivery and routine practices is important for understanding how health facility and service delivery characteristics relate to patient outcomes of interest.

Thank you for your time completing this survey. We are very grateful for your participation.

QUESTIONS	RESPONSES
**1. RESPONDENT INFORMATION**	
**1.1 Name of person completing this survey**	
**1.2 Email address of the person completing the survey**	
**1.3 Please enter the date this survey is being completed**	__ __/__ __/ 2023 (DD / MM / YYYY)
**1.4 What is your title?**	☐ Head Clinician/Clinical Officer In-Charge<br>☐ Other clinician<br>☐ Site Manager<br>☐ Site Data Manager<br>☐ Head Nurse<br>☐ Other (specify) ____________________________
**2. PATIENT POPULATION**	
**2.1 How would you describe the area of residence of the population served by this health facility's HIV clinic(s)?** *Select one response only.*	☐ Predominantly urban<br>☐ Predominantly rural<br>☐ Mixed urban/rural
**2.2 What are the age limits for patients eligible to receive care at this HIV clinic?** *Record minimum and maximum age eligibility limits for HIV patients at this clinic. If there is no minimum age eligibility limit enter 0. If there is no maximum age eligibility limit, enter 99.*	Minimum age limit (years):__________<br>Maximum age limit (years):__________

15. CANCER SCREENING PROVIDED TO PATIENTS WITH HIV				
**15.1 Which of the following cancer screenings were routinely performed during follow-up visits for enrolled patients with HIV.** *Select one best response.*	**Provided in HIV Clinic**	**In same health facility (but not at HIV clinic)**	**Only offsite (referral)**	**Not available**
a. Cervical cancer screening by visual inspection	☐	☐	☐	☐
b. Cervical cancer screening by Pap smear	☐	☐	☐	☐
c. Molecular cervical HPV testing (self-collected or provider-collected swab)	☐	☐	☐	☐
d. Anal pap test	☐	☐	☐	☐
e. High-resolution anoscopy	☐	☐	☐	☐
f. Ultrasound for liver disease management and liver cancer screening	☐	☐	☐	☐
g. Other liver cancer screening tests (such as CT scan or serum alpha fetal protein measurements)	☐	☐	☐	☐
h. Breast exam by provider	☐	☐	☐	☐
i. Breast mammography or ultrasound	☐	☐	☐	☐
j. Colon cancer screening (such as fecal occult blood tests or colonoscopy)	☐	☐	☐	☐
k. Lung cancer screening (such as x-ray or CT scan)	☐	☐	☐	☐
l. Prostate cancer screening by laboratory test	☐	☐	☐	☐

**15.2 What types of patients are routinely screened for the following cancers?** *Multiple answers allowed. Check all that apply or select "None"*	**Male sex at birth**	**Female sex at birth**	**Patients in specific age groups**	**Patients with specific risk factors**	**Other patient groups**	**None / no patients routinely screened**
a. Cervical cancer	☐	☐	☐	☐	☐	☐
b. Anal cancer	☐	☐	☐	☐	☐	☐
c. Liver cancer	☐	☐	☐	☐	☐	☐
d. Breast cancer	☐	☐	☐	☐	☐	☐
e. Colon cancer	☐	☐	☐	☐	☐	☐
f. Lung cancer	☐	☐	☐	☐	☐	☐
g. Prostate cancer	☐	☐	☐	☐	☐	☐

**15.3 What clinic barriers limit or impede routine screening for the following cancers to patients with HIV at this health facility?** *Multiple answers allowed. Check all that apply or select "None."*	**Lack of trained staff to perform screening**	**Lack of equipment to perform screening**	**Lack of standardized or national screening guidelines to inform clinic policy**	**Other**	**None / no barriers for screening**
a. Cervical cancer	☐	☐	☐	☐	☐
b. Anal cancer	☐	☐	☐	☐	☐
c. Liver cancer	☐	☐	☐	☐	☐
d. Breast cancer	☐	☐	☐	☐	☐
e. Colon cancer	☐	☐	☐	☐	☐
f. Lung cancer	☐	☐	☐	☐	☐
g. Prostate cancer	☐	☐	☐	☐	☐

Supplementary Table 2: Site-level characteristics and reported availability of cancer screening services at HIV treatment sites either at the HIV clinic, in the same facility, or off-site in the IeDEA consortium, 2023 (n=220). Count and row percentages shown.

	Cervical Cancer Screening						***Anal Cancer Screening***					
	**Cervical cytology**			**HPV testing**			**Anal cytology**			**High-resolution anoscopy**		
	HIV clinic	*Same Facility*	*Off site*	*HIV clinic*	*Same Facility*	*Off site*	*HIV clinic*	*Same Facility*	*Off site*	*HIV clinic*	*Same Facility*	*Off site*
Overall (n=220)	68 (30.9%)	50 (22.7%)	49 (22.3%)	58 (26.4%)	40 (18.2%)	42 (19.1%)	38 (17.3%)	12 (5.5%)	55 (25%)	17 (7.7%)	18 (8.2%)	68 (30.9%)
IeDEA region												
Asia-Pacific (n=38)	14 (36.8%)	11 (29%)	7 (18.4%)	17 (44.7%)	11 (29%)	6 (15.8%)	6 (15.8%)	4 (10.5%)	11 (29%)	6 (15.8%)	4 (10.5%)	13 (34.2%)
CCASAnet (n=8)	4 (50%)	1 (12.5%)	3 (37.5%)	1 (12.5%)	1 (12.5%)	4 (50%)	3 (37.5%)	0	2 (25%)	2 (25%)	3 (37.5%)	2 (25%)
Central Africa (n=24)	3 (12.5%)	9 (37.5%)	6 (25%)	4 (16.7%)	5 (20.8%)	7 (29.2%)	0	1 (4.2%)	9 (37.5%)	0	0	9 (37.5%)
East Africa (n=75)	9 (12%)	14 (18.7%)	24 (32%)	8 (10.7%)	5 (6.7%)	17 (22.7%)	0	3 (4%)	17 (22.7%)	0	1 (1.3%)	19 (25.3%)
NA-ACCORD (n=34)	21 (61.7%)	12 (35.3%)	1 (2.9%)	16 (47.1%)	16 (47.1%)	1 (2.9%)	24 (70.6%)	2 (5.9%)	8 (23.5%)	9 (26.5%)	9 (26.5%)	15 (44.1%)
Southern Africa (n=31)	17 (54.8%)	2 (6.5%)	7 (22.6%)	8 (25.8%)	1 (3.2%)	5 (16.1%)	5 (16.1%)	2 (6.5%)	4 (12.9%)	0	1 (3.2%)	6 (19.4%)
West Africa (n=10)	0	1 (10%)	1 (10%)	4 (40%)	1 (10%)	2 (20%)	0	0	4 (40%)	0	0	4 (40%)
Residence of population served												
Urban (n=94)	39 (41.5%)	25 (26.6%)	14 (14.9%)	35 (37.2%)	25 (26.6%)	15 (16%)	30 (31.9%)	5 (5.3%)	27 (28.7%)	13 (13.8%)	13 (13.8%)	37 (39.4%)
Mixed Urban/Rural (n=79)	21 (26.6%)	18 (22.8%)	16 (20.3%)	20 (25.3%)	14 (17.7%)	13 (16.5%)	7 (8.9%)	6 (7.6%)	17 (21.5%)	4 (5.1%)	4 (5.1%)	20 (25.3%)
Rural (n=47)	8 (17%)	7 (14.9%)	19 (40.4%)	3 (6.4%)	1 (2.1%)	14 (29.8%)	1 (2.1%)	1 (2.1%)	11 (23.4%)	0	1 (2.1%)	11 (23.4%)
World Bank income designation												
High income (n=55)	34 (61.8%)	14 (25.5%)	4 (7.3%)	32 (58.2%)	18 (32.7%)	3 (5.5%)	28 (50.9%)	3 (5.5%)	14 (25.5%)	12 (21.8%)	12 (21.8%)	22 (40%)
Upper middle income (n=28)	14 (50%)	9 (32.1%)	3 (10.7%)	7 (25%)	8 (28.6%)	4 (14.3%)	8 (28.6%)	3 (10.7%)	3 (10.7%)	5 (17.9%)	3 (10.7%)	8 (28.6%)

Lower middle income (n=82)	12 (14.6%)	10 (12.2%)	31 (37.8%)	9 (11%)	8 (9.7%)	25 (30.5%)	2 (2.4%)	5 (6.1%)	26 (31.7%)	0	3 (3.7%)	29 (35.4%)
Low income (n=55)	8 (14.6%)	17 (30.9%)	11 (20%)	10 (18.2%)	6 (10.9%)	10 (18.2%)	0	1 (1.8%)	12 (21.8%)	0	0	9 (16.4%)

a)Molecular cervical HPV testing by self-collection or provider-collected, b) Liver cancer screening by ultrasound, c) Other liver cancer screening tests include CT scan or serum alpha-fetoprotein measurements, d) Imaging for breast cancer screening included mammography or ultrasound, e) Colon cancer screening included fecal occult blood tests or colonoscopy, f) Lung cancer screening included by x-ray and CT scan, g) Prostate cancer screening by laboratory test. Abbreviations: HRA=High resolution anoscopy

Supplementary Table 3: Adjusted odds of cervical and anal cancer screening from 2017 through 2023 using generalized estimating equations with a logit link function to account for site clustering, rurality, and country income-level by World Bank income designation.

	Cervical Cancer Screening		Anal Cancer Screening	
	Adjusted odds Ratio	95% Confidence Interval	Adjusted odds Ratio	95% Confidence Interval
**Residence of population served**				
Urban	Reference		Reference	
Mixed Urban/Rural	1.05	0.63 – 1.79	0.55	0.35 – 0.87
Rural	0.77	0.44 – 1.33	0.14	0.05 – 0.36
**World Bank income designation**				
High income	Reference		Reference	
Upper middle income	2.19	0.71 – 6.7	0.63	0.36 – 1.08
Lower middle income	0.44	0.23 – 0.84	0.22	0.13 – 0.37
Low income	0.15	0.08 – 0.28	0.06	0.03 – 0.14
**Cancer Screening Availability, by calendar year**	1.16	1.07 – 1.27	0.91	0.84 – 0.99

Supplementary Figure 1: Flow diagram of survey respondents for each survey. Survey 1 was conducted in 2017, survey 2 in 2020 and survey 3 in 2023.

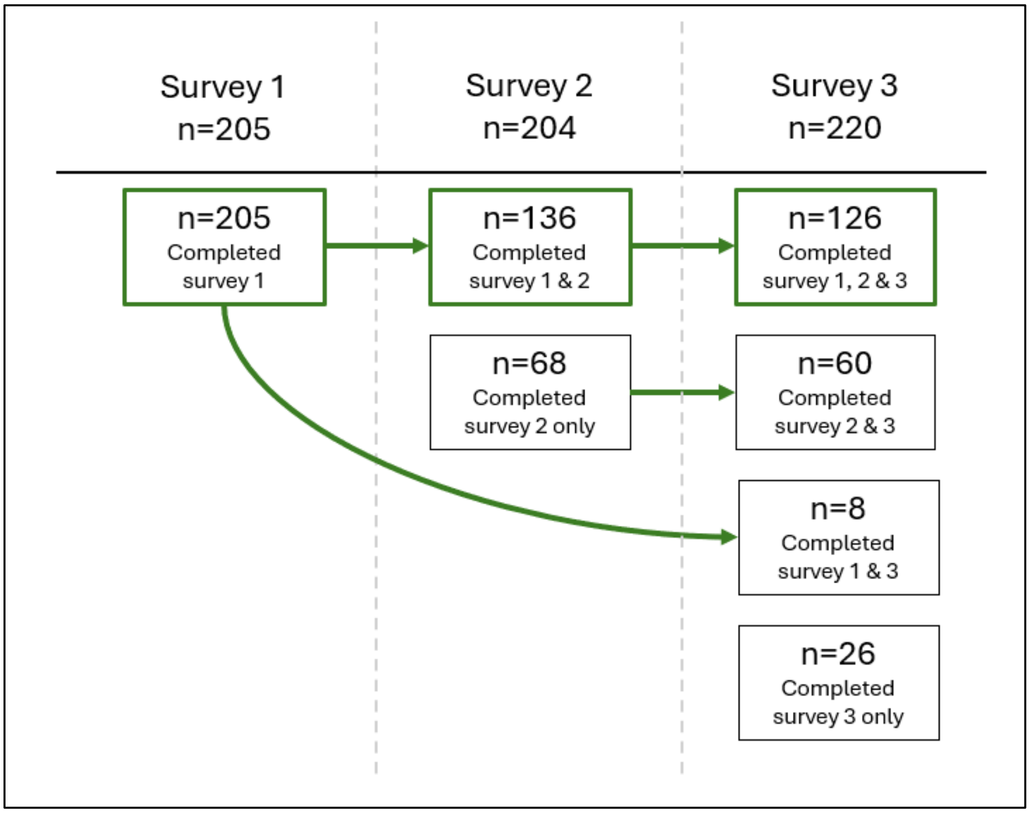

Supplement: Supplementary file 1 — Table S1: Questions 1, 2, and 15 of the IeDEA 2023 site assessment survey. Table S2: Site‐level characteristics and reported availability of cancer screening services at HIV treatment sites either at the HIV clinic, in the same facility, or off‐site in the IeDEA consortium, 2023 (n = 220). Count and row percentages shown. Table S3: Adjusted odds of cervical and anal cancer screening from 2017 through 2023 using generalized estimating equations with a logit link function to account for site clustering, rurality, and country income‐level by World Bank income designation. Figure S1: Flow diagram of survey respondents for each survey. Survey 1 was conducted in 2017, survey 2 in 2020 and survey 3 in 2023. [file IJC-159-1477-s001.pdf]
